# Single Intranasal Administration of Ucn3 Affects the Development of PTSD Symptoms in an Animal Model

**DOI:** 10.3390/ijms252211908

**Published:** 2024-11-06

**Authors:** Andrej Tillinger, Alexandra Zvozilová, Mojmír Mach, Ľubica Horváthová, Lila Dziewiczová, Jana Osacká

**Affiliations:** 1 Biomedical Research Center of the Slovak Academy of Sciences, Institute of Experimental Endocrinology, 845 05 Bratislava, Slovakia; 2 Centre of Experimental Medicine of the Slovak Academy of Sciences, Institute of Experimental Pharmacology & Toxicology, 841 04 Bratislava, Slovakia; 3 Department of Animal Physiology and Ethology, Faculty of Natural Sciences, Comenius University Bratislava, 842 15 Bratislava, Slovakia

**Keywords:** post-traumatic stress disorder, anxiety-like behavior, CRH system, urocortin 3, HPA axis

## Abstract

Post-traumatic stress disorder (PTSD) is a multifactorial psychological disorder that affects different neurotransmitter systems, including the central CRH system. CRH acts via the CRHR1 and CRHR2 receptors, which exert opposite effects, i.e., anxiogenic or anxiolytic. The aim of this work was to investigate how intranasal administration of the CRHR2-specific agonist urocortin 2 (Ucn2) or urocortin 3 (Ucn3) affects manifestations of PTSD in a single prolonged stress (SPS) animal model of PTSD. Elevated plus maze (EPM) and open field (OF) tests were used to assess anxiety-like behavior. Changes in the gene expressions of *CRH*, *CRHR1*, *CRHR2*, glucocorticoid receptor (*GR*), and *FKBP5* were measured in brain regions (BNST, amygdala, and PVN) responsible for modulating the stress response. The SPS animals spent less time in the OF central zone and were less mobile than the controls; however, the Ucn3 treatment reversed this effect. SPS decreased the *GR* and *FKPB5* mRNA levels in the PVN. Ucn3 suppressed the effect of SPS on *FKBP5* mRNA expression in the PVN and increased *FKBP5* mRNA in the BNST and PVN compared to the stressed animals. We demonstrate that Ucn3 has the potential to ameliorate anxiety-like behavior in SPS animals and also to affect the neuroendocrine system in the BNST and PVN. In addition, we confirm the important role of CRHR2 signaling in mediating the stress response.

## 1. Introduction

Post-traumatic stress disorder (PTSD) is classified as a trauma- and stress-related psychiatric disorder. It is characterized by hypervigilance, heightened startle reactivity, hyperarousal, increased anxiety, the generalization of fearful stimuli, and deficits in fear extinction (Diagnostic and statistical manual of mental disorders: DSM-5, 5th edition, 2013).

The precise neurobiological processes behind PTSD pathology have not been fully clarified; moreover, no general mechanism for its development has been proposed. Consequently, the treatment of patients with PTSD is primarily focused on suppressing the symptoms and stimulating functional recovery using a combination of both psychological and pharmacological tools. Pharmacotherapy is mostly used for the treatment of specific symptoms or comorbidities associated with PTSD, including anxiety, depression, psychosomatic symptoms, and sleep disturbances [1]. In addition to conventional pharmacotherapy like antidepressants, antipsychotics, antiepileptics, adrenergic blockers etc., neuropeptides, such as corticotropin-releasing hormone (CRH), vasopressin (AVP), oxytocin (OXY), and neuropeptide Y (NPY), have been suggested as a potential novel therapy for PTSD. Some of these neuropeptides, including OXY and NPY, are already in clinical trials [1,2]. Given their involvement in the regulation of the stress response of an organism and the associated anxiety-related behaviors and psychiatric disorders, neuropeptides represent a promising target for the development of novel treatments for stress- and anxiety-related disorders and are within the scope of researchers. Moreover, besides developing treatments for symptoms related to PTSD, there has also been a shift in research focused on the development of interventions targeting the specific time frame following exposure to traumatic events. The so-called “golden hour” is defined as a limited temporal window during which the stress response is triggered by trauma and can be modified, subsequently affecting the development of neuro-psychological symptoms associated with PTSD [3].

Nevertheless, the practical use of neuropeptides in the treatment of psychological disorders has some drawbacks that have to be considered, like their short half-life, poor blood–brain barrier penetration, broad receptor action, and unstable metabolism. To overcome some of these obstacles, an intranasal (IN) route of delivery is considered a highly practical and convenient solution, with OXY and NPY already in testing [2].

As previously stated, PTSD is a stress-related disorder that affects different neurotransmitter systems, including the central CRH system, which is responsible for regulating the stress response [4,5]. Following the activation of the neuroendocrine stress response pathway in the brain, CRH is released from the paraventricular nucleus (PVN) of the hypothalamus. CRH plays a pivotal role in the regulation of the hypothalamic–pituitary–adrenal (HPA) axis and the subsequent modulation of glucocorticoid plasma levels (cortisol in humans and corticosterone in rodents), which maintain the homeostasis of the organism exposed to stressors. In addition to the PVN, CRH expression was also found in extrahypothalamic brain nuclei, including the amygdala (Amy), the bed nucleus of the stria terminalis (BNST), and the hippocampus. This suggests that CRH plays an integrative role in the neuroendocrine, behavioral, and metabolic responses to stressors. Deregulation of the stress response can lead to a range of severe psychological outcomes. Chronic activation of the CRH system has been associated with various stress-related psychiatric disorders, including anxiety, anorexia nervosa, depression, and PTSD (for a review, see [6,7]). In addition to CRH, there are also mammalian CRH-like paralogs called urocortins (urocortin 1 (Ucn1), urocortin 2 (Ucn2), and urocortin 3 (Ucn3)), which together belong to CRH-peptide family. These peptides act by binding to two different G protein-coupled receptors, particularly the CRH receptor type 1 (CRHR1) and CRH receptor type 2 (CRHR2). While CRH is a high-affinity ligand for CRHR1, Ucn1 binds with equal affinity to both receptors, and Ucn2 and Ucn3 are the preferred ligands for CRHR2 [8].

Elevated levels of CRH were observed in the brains of individuals affected by PTSD [9]. Altered function of the HPA axis is characteristic of patients with PTSD, too [10,11]. The over-expression of central CRH is associated with an anxiogenic behavioral phenotype, which is proposed to be mediated through the CRHR1 signaling pathway. This hypothesis is supported by animal experiments. The CRH/CRHR1 countering effect is believed to be mediated through the CRHR2 signaling pathway, which is responsible for the anxiolytic effect, stress recovery, and maintaining physiological and psychological homeostasis. Animals with *CRHR2* knockout or that of its ligands (*Ucn2* KO and *Ucn3* KO) display increased anxiety, corticosterone response, and impaired stress recovery (for a review, see [5,6]). On the other hand, this simplistic, dualistic mechanism has been challenged by recent findings indicating that the CRH stress response mechanism is more complex and depends on the stressor type and brain regions involved in the stress response [12]. Nevertheless, CRHR1, especially its antagonism, has been proposed as a promising target for the development of treatments for psychiatric disorders such as PTSD and generalized anxiety disorder. Unfortunately, clinical trials of CRHR1 antagonists were mostly unsuccessful and failed to meet the expectations [13]. Conversely, CRHR2 signaling in anxiety-related disorders has not been studied as thoroughly [14]. Nevertheless, there are observations that indicate the importance of CRHR2 neurons, especially in the bed nucleus of the stria terminalis in the development of PTSD [15,16,17], stress response, and recovery [18]. Bagosi and colleagues, in their recent works, demonstrated that the administration of specific CRHR2 agonists, Ucn2 or Ucn3, at the central level (ICV), resulted in a dose- and time-dependent production of hypothalamic CRH. This, in turn, led to a modulation of the HPA axis activity, which subsequently affected corticosterone plasma levels [19,20]. Moreover, they also showed that the central administration of Ucn2 or Ucn3 has the potential to ameliorate anxiety- and depressive-like behavior [21].

Based on up-to-date knowledge, the hypothesis behind our study is that intranasal administration of CRHR2 agonists (Ucn2 and Ucn3) may potentially prevent the development of PTSD-like manifestations at the behavioral and molecular levels. The present study examines the alterations in anxiety-like behavior and the expressions of selected genes associated with the CRH system and HPA axis activity (*CRH*, *CRHR1*, *CRHR2*, glucocorticoid receptor (*GR*), and *FKBP5*) in the brain regions responsible for anxiety, fear processing, and the stress response, specifically the BNST, Amy, and PVN in a single prolonged stress (SPS) animal model of PTSD after acute intranasal application of Ucn2 or Ucn3.

## 2. Results

### 2.1. Effects of Intranasal CRHR2 Agonist Administration on the Development of Behavioral PTSD Manifestation

**Figure 1:**
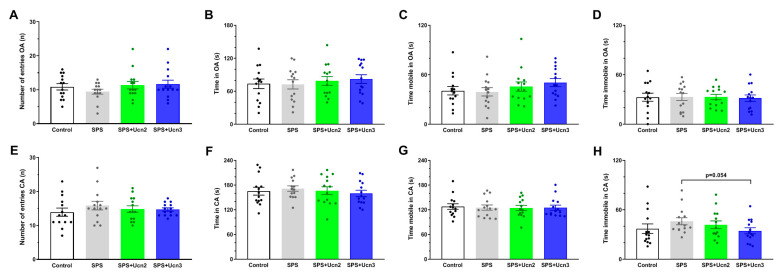
The effects of intranasal CRHR2 agonist administration on anxiety-like behavior. The results of the elevated plus maze (EPM) behavioral measurements in the open arm (OA; (**A**–**D**)) and in the closed arm (CA; (**E**–**H**)). The data are presented as averages of the experimental group (mean ± SEM, *n* = 14 animals per group).

Animals underwent elevated plus maze (EPM) behavioral testing 7 days after SPS protocol and an open field (OF) test 48 h after the EPM test. The EPM test was performed to examine whether CRHR2 agonist (Ucn2 or Ucn3) treatment could prevent the development of anxiety-like behavior in the SPS animals compared to the vehicle-treated ones. Exposure to SPS did not significantly decrease the time that the animals spent in the open arms of the EPM. Intranasal treatment with Ucn2 or Ucn3 did not affect the observed parameters either. The IN Ucn3 treatment of the SPS animals resulted in decreased immobility in the closed arms (CAs), though this change was not significant (t(14) = 245.500, *p* = 0.054; Figure 1H).

**Figure 2:**
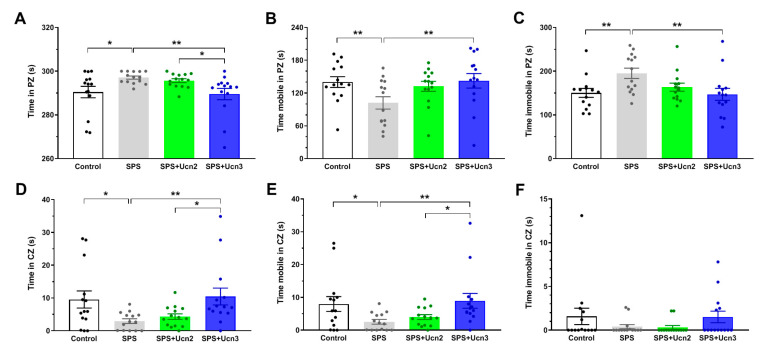
The effects of intranasal CRHR2 agonist administration on anxiety-like behavior. The results of the open field (OF) behavioral measurements in the peripheral zone (PZ; (**A**–**C**)) and in the central zone (CZ; (**D**–**F**)). The data are presented as averages of the experimental group (mean ± SEM, *n* = 14 animals per group). * *p* < 0.05, ** *p* < 0.01.

The results of the OF test, performed 48 h after the EPM test, are much more promising and confirm the trends seen in the EPM test. In the OF test, SPS led to a significant decrease in the time that the animals spent in the central zone (CZ) (F(3,52) = 3.7989, *p* < 0.05; Figure 2D), and they also spent significantly more time in the peripheral zone (PZ) (F(3,52) = 3.7989, *p* < 0.05; Figure 2A) compared to the control animals. Intranasal treatment of the SPS animals with Ucn3 (SPS + Ucn3) had a protective effect, resulting in the animals spending more time in the CZ (F(3,52) = 3.7989, *p* < 0.01) and less time in the PZ (F(3,52) = 3.7989, *p* < 0.01) compared to the VEH-treated SPS animals (Figure 2 A,D). Furthermore, the Ucn3 treatment was also more effective than the Ucn2 treatment (F(3,52) = 3.7989; time in the CZ, *p* < 0.05; time in the PZ, *p* < 0.05; Figure 2A,D). We observed the same results when comparing mobility in the CZ, where the SPS animals were less mobile than the controls (F(3,52) = 3.3305, *p* < 0.05), the Ucn3 application suppressed the SPS effect (F(3,52) = 3.3305, *p* < 0.01), and again, the Ucn3 treatment was more effective than the Ucn2 (F(3,52) = 3.3305, *p* < 0.05; Figure 2E). Based on these results, we can assume that the IN Ucn3 treatment had a protective effect and prevented the development of anxiety-like behaviors in the animals exposed to SPS.

### 2.2. Effects of Intranasal CRHR2 Agonist Administration on Plasma Corticosterone Levels in SPS Animals

**Figure 3:**
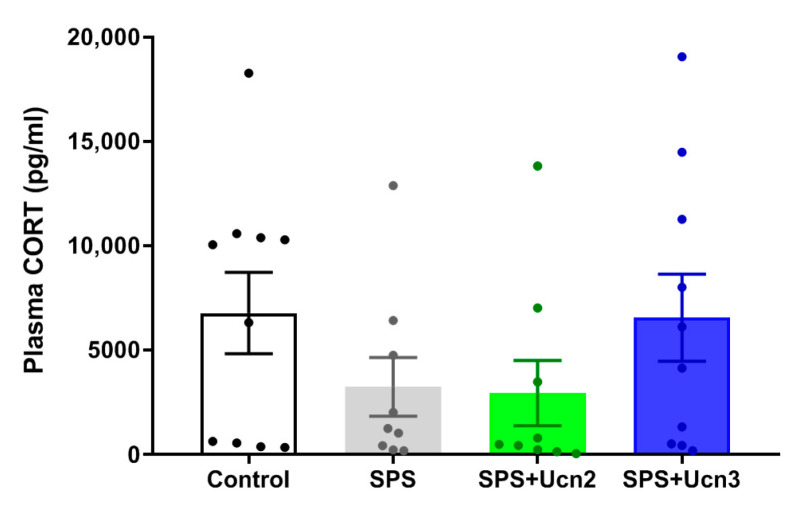
The effects of the intranasal administration of CRHR2 agonists on corticosterone (CORT) plasmatic levels. The data are presented as averages of the experimental group (mean ± SEM, *n* = 9–10 animals per group).

The plasma corticosterone (CORT) levels were measured in the trunk blood of animals that were decapitated 72 h after the OF test (12 days after the SPS procedure) to avoid any stress carryover. We found a decrease in the plasma CORT levels after SPS, which was prevented by the Ucn3 treatment, but these changes were not significant due to higher variability among the samples (Figure 3). Nevertheless, even though these changes were not significant, they complement the overall positive outcome of the IN Ucn3 treatment, together with the other results.

### 2.3. Changes in Gene Expressions in Brain Areas Responsible for Stress Recovery and Anxiety Handling After Intranasal CRHR2 Agonist Administration

To evaluate the effects of CRHR2 agonist administration on the development of PTSD-like manifestations in the SPS animal model, we measured the mRNA levels of *CRH*, *CRHR1*, *CRHR2*, *GR*, and *FKBP5* in the brain regions involved in stress recovery and fear and anxiety processing, i.e., the BNST, Amy, and PVN.

**Figure 4:**
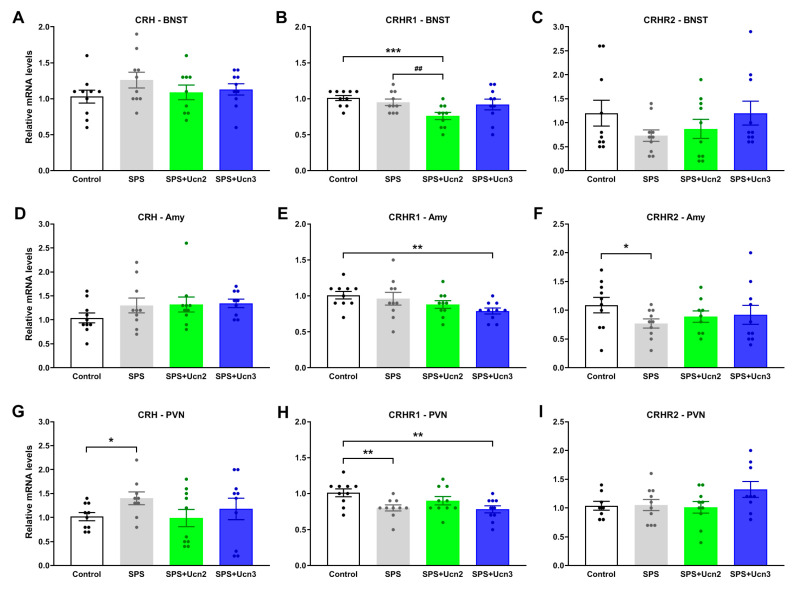
Changes in the gene expressions of *CRH* (**A**,**D**,**G**), *CRHR1* (**B**,**E**,**H**), and *CRHR2* (**C**,**F**,**I**) in selected brain regions involved in stress response mechanisms (BNST, Amy, and PVN). The data are presented as the fold changes relative to the control, taken as 1 (mean ± SEM, *n* = 8–10 animals per group). *t*-test: * *p* < 0.05, ** *p* < 0.01, *** *p* < 0.001 vs. control, ## *p* < 0.01 SPS vs. SPS + Ucn2.

In the PVN, exposure to SPS significantly increased the *CRH* mRNA levels (t(17) = −2.419, *p* < 0.05; Figure 4G), and treatment with Ucn2 and Ucn3 showed trends to prevent this increase (Figure 4G). In the BNST and AMY, we did not observe any changes in the *CRH* mRNA levels in either the SPS or SPS + Ucn2/3 experimental groups (Figure 4A,D).

In the PVN, we observed decreased levels of *CRHR1* mRNA in the SPS and SPS + Ucn3 animals compared to the controls (t(18) = 3.204, *p* < 0.01 and t(18) = 3.606, *p* < 0.01, respectively; Figure 4H). The *CRHR1* mRNA levels were also significantly lower in the BNST of the SPS + Ucn2 animals compared to the unstressed controls (t(18) = 3.927, *p* < 0.001) and SPS animals (t(18) = 2.994, *p* < 0.01; Figure 4B). Ucn3 administration to the SPS animals suppressed the *CRHR1* mRNA levels in the Amy compared to the controls (t(18) = 3.371, *p* < 0.01; Figure 4E).

The *CRHR2* mRNA levels were significantly lower only in the Amy of the SPS animals compared to the unstressed controls (t(18) = 2.114, *p* < 0.05; Figure 4F). We also measured changes in the gene expressions of *GR* and *FBKB5*, which are considered markers of HPA axis activity and are closely associated with PTSD development, particularly polymorphisms in the *FKBP5* gene [22,23].

**Figure 5:**
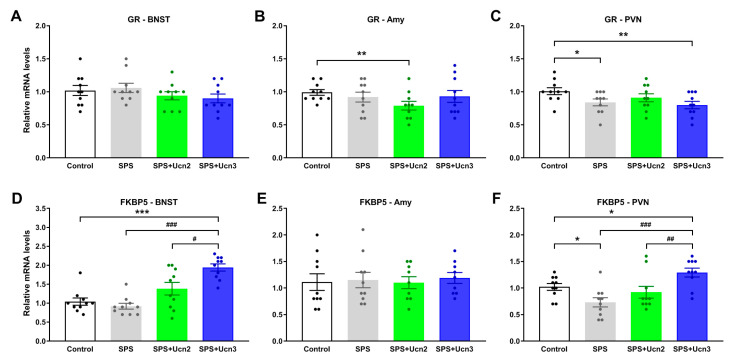
Changes in the gene expressions of the glucocorticoid receptor (*GR*; (**A**–**C**)) and *FKBP5* (**D**–**F**) in selected brain regions involved in stress response mechanisms (BNST, Amy, and PVN). The data are presented as fold changes relative to the control, taken as 1 (mean ± SEM, *n* = 8–10 animals per group). *t*-test: * *p* < 0.05, ** *p* < 0.01, *** *p* < 0.001 vs. control, # *p* < 0.05, ## *p* < 0.01, ### *p* < 0.001 vs. SPS + Ucn3.

The *GR* mRNA levels were significantly lower in the PVN of the SPS (t(18) = 2.383, *p* < 0.05) and SPS + Ucn3 (t(18) = 2.858, *p* < 0.01) experimental groups than in the controls (Figure 5C). Ucn2 treatment of the SPS animals resulted in a significant decrease in *GR* gene expression in the Amy compared to the unstressed control animals (t(18) = 2.979, *p* < 0.01, Figure 5B). No changes in *GR* expression were observed in the BNST.

The most significant and obvious changes were observed in the gene expressions of *FKBP5* in the PVN and BNST. In the PVN, exposure to SPS significantly decreased (t(18) = −2.699, *p* < 0.05) and the Ucn3 treatment of SPS animals significantly increased the *FKBP5* mRNA levels compared to the control animals (t(10) = 136, *p* < 0.05; Figure 5F). The SPS + Ucn3 animals also exhibited higher *FKBP5* mRNA levels than the SPS (t(18) = 4.491, *p* < 0.001; Figure 5F) and SPS + Ucn2 (t(18) = 2.919, *p* < 0.01; Figure 5F) animals. In the BNST of the SPS + Ucn3 animals, we observed increased *FKBP5* mRNA levels compared to the control (t(18) = −6.727, *p* < 0.001), SPS (t(18) = −8.255, *p* < 0.001) and SPS + Ucn2 animals (t(10) = 72, *p* < 0.05; Figure 5D). No changes in the gene expression of *FKBP5* were observed in the Amy (Figure 5E).

### 2.4. Changes in CRH, cAMP Response Element-Binding Protein (CREB) and Phosphorylated CREB (pCREB) Proteins in Brain Areas Responsible for Stress Recovery and Anxiety Handling After Intranasal CRHR2 Agonist Administration

We examined the expressions of CRH, CREB, and pCREB by immunohistochemistry. Transcription factor CREB has been shown to be involved in the activation of the CRH gene, and a functional correlation between the presence of the active phosphorylated form of CREB, i.e., pCREB, and CRH, under stressful conditions has also been demonstrated [24,25]. The mechanism of suppression of CRH biosynthesis by glucocorticoids may involve the inhibition of CREB phosphorylation [26].

**Figure 6:**
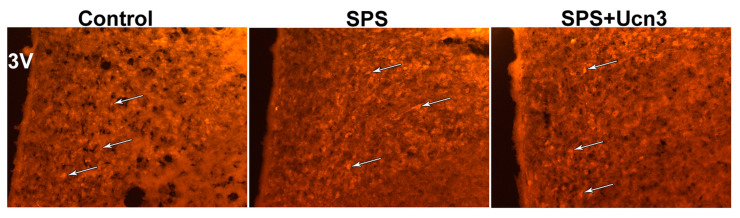
Representative images of immunofluorescence staining with anti-CRH in the PVN. 3V—3rd ventricle. White arrows indicate CRH-immunopositive neurons. Magnification ×200.

The intensity of CRH immunofluorescence in the PVN was evaluated as the mean intensity. We detected more CRH-immunopositive neurons in the SPS animals than in the controls, but the differences in mean intensity were not statistically significant (1.7 ± 1 AU for the control vs. 5.1 ± 1.2 AU for the SPS animals). No significant differences were found between the SPS (5.1 ± 1.2 AU) and SPS + Ucn2 (4.9 ± 1.9 AU) or SPS + Ucn3 (4.9 ± 0.9 AU) groups (Figure 6). In the BNST and Amy, no significant differences in CRH immunopositivity were observed between the experimental groups.

**Figure 7:**
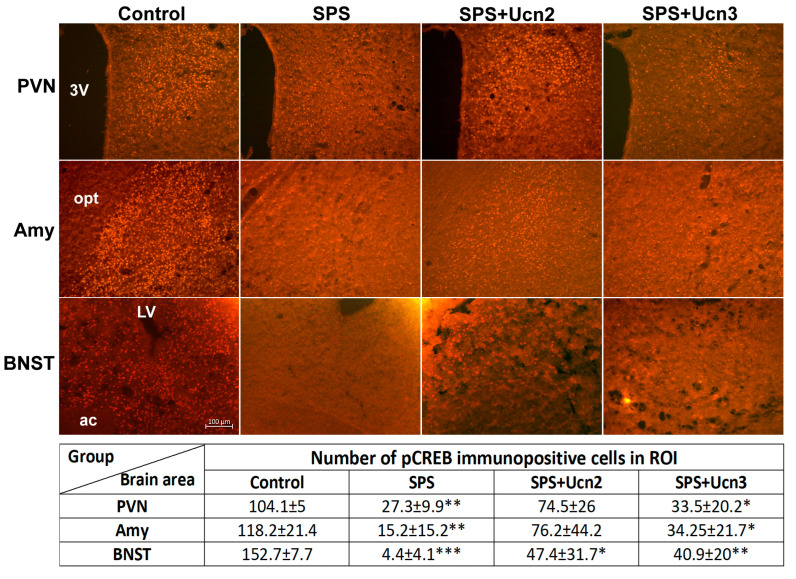
Representative images of immunofluorescence staining with anti-pCREB in the brain regions involved in stress response mechanisms (PVN, Amy, and BNST). The table shows the number of pCREB-immunopositive cells in the ROI (region of interest). 3V—3rd ventricle, ac—anterior commissure, LV—lateral ventricle, opt—optic tract. *t*-test: * *p* < 0.05, ** *p* < 0.01, *** *p* < 0.001 vs. control.

CREB-immunopositive cells were scattered throughout the PVN, BNST, and Amy, with no significant differences among the experimental groups. In the PVN of the SPS animals, the CREB-immunopositive cells were mainly concentrated in the parvocellular part. The highest number of pCREB-immunopositive cells was detected in the control animals. Interestingly, the SPS animals had fewer pCREB-positive cells in the PVN (t(5) = 6.213, *p* < 0.01), BNST (t(6) = 16,964, *p* < 0.001), and Amy (t(6) = 3.925, *p* < 0.01) than the controls (Figure 7). The application of Ucn2 and Ucn3 to the SPS animals only slightly increased the number of pCREB neurons in all three areas examined compared to the SPS animals. However, the SPS + Ucn3 animals had fewer pCREB-immunopositive cells than the controls in all three brain areas (t(5) = 2.911, *p* < 0.05 for PVN, t(6) = 5.215, *p* < 0.01 for the BNST, and t(6) = 2.751, *p* < 0.05 for the Amy), while the SPS + Ucn2 animals had fewer pCREB-immunopositive cells only in the BNST (t(5) = 2.762, *p* < 0.05) (Figure 7).

## 3. Discussion

In our study, we found that a single intranasal administration of the highly selective CRHR2 agonist urocortin 3 prevented the development of behavioral outcomes of PTSD in the SPS animal model. Ucn3 administration to SPS animals increased their locomotor activity and decreased their anxiety-like behavior in the OF test. Interestingly, we did not observe the same effects with another CRHR2 specific agonist, urocortin 2, which was also administered intranasally at the same dose as urocortin 3. Not only did Ucn2 not exert an anxiolytic effect, but its effect was significantly inferior compared to Ucn3. In the EPM test, we did not see any significant effects of any of the CRHR2 agonists used. The discrepancy seen between the EPM and OF test results may be due to the shorter time period after SPS when the behavioral tests were performed. Perhaps a longer habituation period after SPS would have been more beneficial and we would have seen more pronounced behavioral changes. However, there are many unknowns, and to the best of our knowledge, this is the first study to investigate the effects of the intranasal administration of CRHR2 agonist on the development of PTSD-like symptoms in an animal model. Therefore, the novelty of our approach and the challenges posed by the use of neuropeptides in pharmacotherapy are the reasons for a shorter time interval. This also raises the question of the timing of the intervention. While we decided on immediate IN application of the CRHR2 agonist after the SPS protocol, in future experiments, it will be necessary to investigate whether the IN application of the CRHR2 agonist days or weeks after stressful events has the potential to ameliorate manifestations of PTSD symptoms. It was shown that IN NPY administration 2 weeks after SPS had a beneficial effect on PTSD behavioral outcomes [27]. This also raises the question of where the borderline is between preventing the development of PTSD symptoms and treating PTSD symptoms. In our study, we aimed at the so-called “golden hour”, a putative time window after the exposure of an organism to stressful events when a pharmacological intervention has the potential to modify the central stress response of the organism and thereby affect/prevent the development of PTSD manifestations [3].

Nevertheless, the results obtained from the behavioral tests suggest that IN Ucn3 administration has the potential to prevent the development of anxiety-like behavior in animal models of PTSD. Similarly, antidepressant and anxiolytic-like effects of Ucn3 have also been shown after ICV administration to mice and rats [28,29,30,31]. On the other hand, we did not observe any changes in the behavioral manifestations of the SPS animals after the Ucn2 treatment. Previous studies have shown that the effect of Ucn2 on anxiety-like behavior is highly variable and differs depending on the level of stress or the brain regions involved [32,33]. In the nicotine withdrawal model, ICV administration of Ucn2 and Ucn3 ameliorated anxiety- and depressive-like behavior in mice, but it is important to note that behavioral testing was performed 30 min after the ICV application [21]. The results from another study also confirm the role of the CRHR2 signaling pathway in the modulation of defensive behavior, which was blocked by the CRHR2 specific antagonist in the ventromedial hypothalamus (VMH), but at the same time, there was no effect after Ucn2 administration to the VMH [34]. These observations are in agreement with experiments performed mainly in genetically modified animals, which have shown that the anxiogenic/anxiolytic effect of the CRH system is not a question of simple dualism, where the CRHR1 signaling pathway is not strictly anxiogenic and the CRHR2 pathway is strictly anxiolytic, and that behavioral or neuroendocrine changes differ depending on the brain regions and neuronal circuits involved in the stress response [5,12].

Behavioral changes are a consequence of altered brain functions, and as noted above, the pathophysiology of PTSD is associated with altered basal levels and the reactivity of a number of neurotransmitter systems involved in the regulation of the organism’s stress response, including the CRH system. Most have studies suggested that excessive CRH activity or greater responsiveness of CRH to stressors is involved in the etiology of PTSD. Understanding the role of the CRH system in mediating fear processing and anxiety- and stress-related behaviors in psychiatric disorders such as PTSD may provide us with potential methods of pharmacological intervention to reverse these maladaptive processes. There have already been studies based on CRHR1 antagonism as a potential therapy for stress-related disorders, but unfortunately, they have failed before translation into clinical practice [13]. Therefore, in our experimental model of PTSD, we decided to investigate whether the intranasal administration of CRHR2 agonists could prevent the development of not only behavioral changes, but also changes in the gene expressions of different components of the CRH system, i.e., *CRH*, *CRHR1*, and *CRHR2* in the BNST, PVN, and Amy. We focused on these interconnected brain regions because they are all involved in the regulation of organisms’ stress response, and their CRH neurons have been shown to play an important role in defensive behavior, which can serve as a basis for the study of fear- and anxiety-related psychiatric disorders such as PTSD. In addition, CRH neurons in the PVN are responsible for triggering the neuroendocrine stress response of the HPA axis [35]. We found increased *CRH* mRNA levels only in the PVN of the SPS animals; in the BNST and Amy, the increase did not reach statistical significance. Similarly, the Sabban group found increased *CRH* mRNA in the mediobasal hypothalamus of SPS animals [27,36,37], which correlates with the increased CRH levels found in the CSF of PTSD patients [9]. After CRHR2 agonist treatment, the *CRH* mRNA levels in the PVN of the SPS animals reached almost unstressed levels, but this change was not statistically significant. Consistent with the mRNA levels, the Ucn2- and Ucn3-treated SPS animals had fewer CRH-immunopositive cells than the SPS animals. Notably, the *CRHR1* mRNA levels were not elevated in any of the brain regions examined in the SPS animals, which was expected based on its proposed anxiogenic effect and previous data showing elevated *CRHR1* levels in certain brain regions after SPS [36,38]. Another study demonstrated an anxiogenic effect of CRH/CRHR1 when the knockdown of *CRHR1* in the basolateral Amy led to decreased anxiety-like behavior [39], and activation of the CRHR1 signaling pathway in the basolateral Amy and BNST induced anxiety-like behavior [40,41]. Surprisingly, we found significantly decreased *CRHR1* mRNA levels in the PVN and no changes in the BNST and Amy. On the other hand, the Ucn2 treatment decreased the *CRHR1* mRNA levels in the BNST of the SPS animals, and the Ucn3 treatment decreased the *CRHR1* mRNA levels in the Amy and PVN. Changes in the *CRHR2* mRNA levels were not clearly conclusive; we saw a decrease in the Amy in the SPS animals and only a slight increase in expression after the Ucn3 treatment in the BNST and PVN. A previous study also showed that *CRHR2* expression after SPS was brain region-specific, as its elevated levels were found in the mPFC but not in the hippocampus or Amy [42].

CREB has been shown to be necessary for stress-related increases in CRH transcription, and the overexpression of CREB in the central Amy CRH neurons increases fear expression and may blunt fear extinction [43]. Therefore, we also detected the expression of CREB and pCREB in the above-mentioned brain areas (BNST, PVN, and Amy). We observed decreased pCREB immunoreactivity in the SPS animals in all the brain areas studies compared to the controls, and Ucn2 and Ucn3 administration caused its slight increase. Changes in pCREB expression were shown to be time- and brain area-dependent. In the predator stress model of PTSD, elevated pCREB was detected in the supramammillary nucleus and dorsal periaqueductal gray as early as 7 days after stress exposure. However, decreased pCREB was found in the Amy 24 h after stress [44]. This may explain the reduced pCREB levels observed in the SPS group.

The HPA axis, the main stress hormone system, plays an important role in regulating the organism’s stress response, and its deregulation is a known feature of PTSD. In general, PTSD is associated with increased CRH levels [9,45,46] and lower or unchanged cortisol levels in patients [47,48]. In our experiment, we observed a decrease in the plasma levels of CORT, similar to observations from another study using the SPS model of PTSD [49]. The Ucn2 treatment of the SPS animals did not affect the plasma CORT levels, but after the Ucn3 treatment, there was an inhibition of the SPS-induced decrease in the plasma CORT levels. Unfortunately, these changes were not statistically significant but were in agreement with the positive effect of the IN Ucn3 treatment observed in the results of the behavioral tests. Previously, it has been shown that ICV administration of Ucn3 has the potential to activate the HPA axis and increase plasma ACTH and CORT levels in a dose-dependent manner [19,50]. This suggests that Ucn3, as a highly selective CRHR2 agonist, has the potential to regulate the central stress response mechanisms. However, as already mentioned, it is not unambiguous, and the effect of activation of the CRHR2 signaling pathway in the way of stress recovery depends on several factors, including the severity of the stress and the brain regions affected and the timing and the dose of the CRHR2 agonist used [5]. Glucocorticoid receptors (GRs) play an important role in the function of the HPA axis, as they are directly involved in the negative feedback of corticosterone by blocking CRH in the PVN and terminating the stress response. The proper negative feedback of GRs is crucial for a healthy stress response and their alterations may affect the pathogenesis of mood disorders [51]. The altered sensitivity of GRs is a consistent finding in individuals with PTSD [10,11]. GRs are activated by ligand binding and translocated to the nucleus where they interact with DNA or other transcription factors and affect the transcription of target genes. These processes are regulated by large molecular complexes (molecular chaperones), one of which is FKBP5. When FKBP5 binds to a GR, it decreases its affinity for the ligand and prevents translocation to the nucleus [52]. Because of its pivotal role in regulating GR function, it has also been the focus of PTSD research [22,23]. In our experiment, we found decreased *GR* mRNA levels in the PVN after SPS. Our data are consistent with other studies that have also shown decreased basal mRNA levels of *GR* and *FKBP5* in the mediobasal hypothalamus after SPS [27,36]. Another study found decreased *FKBP5* gene expression after SPS not only in the PVN but also in the Amy [53]. The Ucn2 treatment decreased only *GR* expression in the Amy, but Ucn3 increased *FKBP5* in the BNST and also in the PVN compared to SPS with and without Ucn2 treatment. In the study using RU486 as an early intervention after SPS, increased protein levels of FKBP5 were shown in the hippocampus and hypothalamus [49], which is consistent with the changes induced by the Ucn3 treatment observed in our study. Increased FKBP5 expression suggests decreased GR sensitivity to ligands and the possible inhibition or reduction of the glucocorticoid response element (GRE)-dependent transcription of genes in brain regions responsible for stress and fear/anxiety modulation. Consistent with increased *FKBP5* mRNA levels after Ucn3 application, we found slightly decreased *CRH* mRNA levels in the PVN, but the results in the BNST were less conclusive. Increased expression of *FKBP5* is associated with impaired regulation of HPA axis feedback, and thereby, the extinction of conditioned fear [54]. In addition, polymorphisms in *FKBP5* have been shown to be associated with distinct biological manifestations of PTSD [55].

In conclusion, to the best of our knowledge, this is the first study demonstrating that a single intranasal application of the CRHR2 agonist Ucn3 has the potential to prevent the development of anxiety-like behaviors in an SPS animal model of PTSD. The results also suggest that the CRH neurotransmitter system in the PVN, BNST, and Amy does not appear to be the primary target of Ucn3, mediating its anxiolytic effect. Nevertheless, we have shown that IN Ucn3 administration modulates HPA axis function. Our data support previous observations that neuropeptides may have potential for the treatment of PTSD, and that intranasal application is a promising way to deliver the active substance to the brain [2].

## 4. Materials and Methods

### 4.1. Animals

Male Sprague–Dawley rats (176–200 g, Charles River, Sulzfeld, Germany) were used in our experiment. Prior to the experiment, the animals were acclimatized for 10 days in the animal facility room to avoid stress carryover. The animals were housed with four per cage in a controlled environment (22 ± 2 °C, 12 h light/dark cycle, lights on at 7 a.m.). Food and water were provided ad libitum. All experimental procedures were performed in accordance with the Council Directive 2010/63EU of the European Parliament and the of the Council of 22 September 2010 on the protection of animals used for scientific purposes and approved by the Committee of the State Veterinary and Food Administration of the Slovak Republic (approval protocol number 5007/2022-220).

### 4.2. Experimental Design

**Figure 8:**
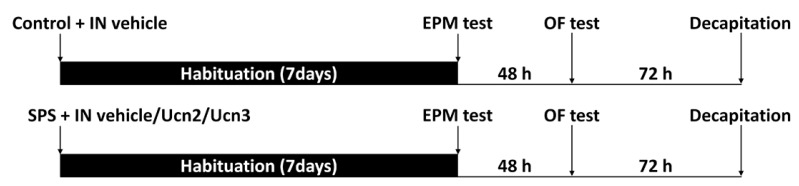
A schematic illustration of the experimental design. The animals were randomly assigned to the four experimental groups which were as follows: control– non-stressed group with intranasal (IN) vehicle; SPS (single prolonged stress)—stressed group with IN vehicle; SPS + Ucn2—stressed group with IN Ucn2; SPS + Ucn3—stressed group with IN Ucn3. After SPS, the animals were left undisturbed in their cages for seven days to develop PTSD symptoms. After 7 days in the habituation period, the animals were tested in the elevated plus maze (EPM), and 48 h after the EPM, in the open field (OF). The animals were sacrificed 72 h after the OF test.

After a 10-day acclimatization period, the animals were randomly assigned to the four experimental groups with 14 animals each. The experimental groups were as follows: **control**—non-stressed group with intranasal vehicle; **SPS**—stressed group with intranasal vehicle; **SPS + Ucn2**—stressed group with intranasal Ucn2 treatment; and **SPS + Ucn3**—stressed group with intranasal Ucn3 treatment. We used a single prolonged stress (SPS) protocol followed by 7 days of sensitization to induce PTSD-like behavior in the animals [56]. For the SPS, the rats were first immobilized for 2 h in plastic restrainers (Flat Bottom Rodent Holders, RSTR544, Kent Scientific Co., Torrington, CT, USA). Immediately after restraint, the rats were immediately subjected to forced swimming for 20 min in a glass cylinder filled to two-thirds with ±24 °C freshwater. The animals were then dried and allowed to recuperate for 15 min, after which they were exposed to ether vapor until loss of consciousness. During this brief period of unconsciousness, the animals were intranasally (IN) administered with one of the following: (i) vehicle (saline, 0,9% NaCl; B Braun, Melsungen, Germany)—SPS and control groups; (ii) urocortin 2 (20 µg/kg, H-6238, urocortin II (mouse); Bachem, Bubendorf, Switzerland)—SPS + Ucn2 group; (iii) urocortin 3 (20 µg/kg, H-5828, urocortin III (mouse); Bachem, Bubendorf, Switzerland)—SPS + Ucn3 group. The control group was also exposed to ether vapors to receive the IN vehicle. The IN treatment was delivered in the form a 10 µL drop in each nostril using a pipette, with the animal’s head tilted backward to ensure complete inhalation of the solution. The animals were then left undisturbed for seven days, with two per cage, to develop PTSD symptoms prior to behavioral testing (Figure 8). After the 7-day senzibilization period, the animals were tested in the elevated plus maze (EPM), and 48 h after the EPM, in the open field (OF) to avoid any carryover. The experiment was concluded 72 h after the OF test, when the animals were sacrificed. A total of 10 animals from each group were decapitated for blood and tissue sample collection, and 4 animals were transcardially perfused for further immunohistochemical staining.

### 4.3. Behavioral Tests

#### 4.3.1. Elevated Plus Maze

All animals were tested 7 days after the SPS protocol in a cross-shaped elevated plus maze (EPM) made of dark plastic. The open and closed arms of the maze were 50 cm above the ground, 50 cm long, and 10 cm wide. The animals were tested for 5 min. After each individual trial, the maze was wiped with 60% ethanol. Their movements were recorded with a digital camera, and individual sessions were analyzed with ANY-maze Video Tracking System 7.1 (Stoelting Co., Wood Dale, IL, USA) computer software. The analysis included counting the number of entries of individual animals to the open arms (OAs) or closed arms (CAs) of the EPM, measuring the total time the animals spent in the OA or CA, and measuring the time the animals were mobile or immobile in the OA or CA.

#### 4.3.2. Open Field Test

The open field (OF) test was conducted in a 60 × 60 cm area divided into central (22.5 × 22.5 cm) and peripheral areas with 40 cm high walls. Lighting was provided by a lamp mounted above the maze. Each session lasted 5 min and began with the rat being placed in the central area. After each trial, the maze was wiped with 60% ethanol. The movements of the rats were recorded with a digital camera, and the individual sessions were analyzed with ANY-maze Video Tracking System 7.1 (Stoelting Co., Wood Dale, IL, USA) computer software. The analysis included measuring the total time spent by the animals in the central zone (CZ) and peripheral zone (PZ) of the OF and measuring the time the animals were mobile or immobile in the CZ and PZ.

### 4.4. Tissue Collection and Preparation

The animals were euthanized by decapitation or transcardial perfusion. After decapitation, the whole brains were dissected from the animals, immediately frozen on dry ice, and stored at −80 °C for further analysis. Trunk blood from the rats was collected in heparinized tubes and centrifuged at 10,000× *g* for 20 min at 4 °C. The plasma was collected and stored at −80 °C for further analysis.

For transcardial perfusion, the rats were anesthetized with sodium pentobarbital (50 mg/kg, i.p.; SPOFA, Prague, Czech Republic) and transcardially perfused with 60 mL saline containing 450 μL heparin (5000 IU/L; Zentiva, Bratislava, Slovakia), followed by 250 mL fixative containing 4% paraformaldehyde in 0.1 M phosphate buffer (PB, pH 7.4). The removed brains were postfixed in fresh fixative overnight, washed twice in 0.1 M PB, infiltrated with 30% sucrose for 2 days at 4 °C, cut into 30 μm-thick coronal sections using a cryostat (Reichert-Jung, Munich, Germany), and collected in cryoprotectant solution at −20 °C until further immunohistochemical processing.

#### Microdissection of the Brain Areas

The frozen brains were cut into 300 µm-thick coronal sections using a cryostat (Reichert-Jung, Munich, Germany), at −12 °C. The brain sections were placed on microscope slides and fixed by brief warming. Selected areas of the forebrain (BNST, Amy, and PVN) were isolated using the micropunching technique according to the original punching guide atlas [57]. The separated tissues were stored at −80 °C for later analysis.

### 4.5. RNA Isolation and Real-Time PCR

The total RNA was isolated using TRI Reagent^®^RT (MRC, Inc., Cincinnati, OH, USA) according to the manufacturer’s protocol, and the concentrations were quantified using NanoDrop 2000 (Thermo Fisher Scientific, Waltham, MA, USA). Reverse transcription of the RNA was performed using the Revert Aid H minus First Strand cDNA Synthesis kit (Thermo Fisher Scientific, Vilnius, Lithuania) according to the manufacturer’s protocol using an oligo dT primer. Semi-quantitative real-time PCR was performed in a total volume of 25 μL containing 30 ng of template cDNA mixed with 12.5 μL FastStart Universal SYBR Green Master Rox (Roche Diagnostics, Mannheim, Germany), 1 μL specific primer pair set, and water. The sequences of the specific primers used were as follows: ***CRH***—forward: 5′-CGATTCTGATCCGCATGGGT-3′, reverse 5′-CAGCAACACGCGGAAAAAGT-3′; ***CRHR1***—forward: 5′-CAATGTGGCCTGGTGTA GGT-3′, reverse: 5′-GGTGGAGTACGTGAGCACAA-3′; ***CRHR2***—forward: 5′-TGCAACTCATCGA CCACGAA-3′, reverse: 5′-GCAGGGTATGCACCATCCAA-3′; ***GR***—forward: 5′-ACCCGAGGTG TTGTATGCAG-3′, reverse: 5′-CTGAAGCCTGGTATCGCCTTT-3′; ***FKBP5***—forward 5′-ATCCTGG GAGATGGACACCA-3′, reverse: 5′-GCCAGGACACTATCTTCCCG-3′; ***GAPDH***—forward: 5′-TGGACCACCCAGCCCAGCAAG-3′, reverse: 5′-GGCCCCTCCTGTTGTTATGGGGT-3′. Each sample was analyzed on a QuantStudio™ 5 Real-Time PCR System (Applied Biosystems, Waltham, MA, USA) under the following conditions: 1 cycle of 2 min at 50 °C, followed by 1 cycle of 10 min at 95 °C, and then 40 cycles of 15 s at 95 °C and of 1 min at 60 °C. The data were normalized to GAPDH levels and expressed as the relative fold changes as calculated by the ΔΔC_t_ method [58]. Melting curve analysis was performed to confirm the specificity of the amplified products.

### 4.6. Measurement of Plasma Corticosterone

The plasma corticosterone (CORT) levels were measured using an ELISA kit (ADI-900-097, Corticosterone ELISA Kit, Enzo Life Sciences, Farmingdale, NY, USA), according to the manufacturer’s instructions.

### 4.7. Immunohistochemistry

**Figure 9:**
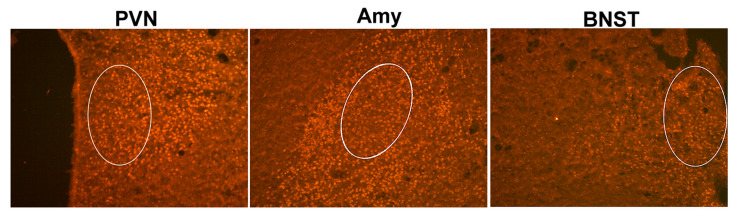
Areas marked with a white circle represent the region of interest (ROI) for counting the pCREB-immunopositive cells. Magnification ×200.

The free-floating sections were washed 3 times for 5 min each in 0.1 M PB (pH = 7.4). For CREB and pCREB, staining sections were incubated in blocking solution (0.1 M PB with 3% NGS and 2% BSA) for 1 h at RT, followed by primary antibody. The primary antibodies were diluted in PB containing 4% NGS, 1% Triton X-100, and 0.1% sodium azide. The primary antibodies were diluted as follows: anti-CREB 1:500 (mouse, 35-0900; Invitrogen, Waltham, MA, USA), anti-pCREB 1:400 (rabbit, MA5-11192; Invitrogen, Waltham, MA, USA), anti-CRH 1:400 (rabbit, Ab8901; Abcam, Cambridge, UK). The sections were incubated with primary antibodies for 48 h at 4 °C. The sections were then washed 3 times for 5 min each in PB and incubated with secondary antibodies for 90 min at RT in the dark. For immunofluorescence, Alexa Fluor 555 (for CRH and pCREB) and Alexa Fluor 488 (for CREB) secondary antibodies were used at a concentration of 1:300 (Invitrogen, Waltham, MA, USA). Finally, the sections were washed in PB 3 times for 5 min each, mounted on adhesive slides, and cover-slipped with Fluoromont (Thermo Fisher Scientific, Waltham, MA, USA) as an anti-fading agent. The sections were visualized with Zeiss AxioImager A1 (Carl Zeiss AG, Jena, Germany). Quantification of the pCREB-immunopositive cells was performed using Fiji/ImageJ 1.54 software in a selected region of interest (oval, ROI, 42 700 µm^2^) that covered most of the examined area. The pCREB-positive cells were counted manually from the ROI in the parvocellular part of the PVN, Amy, and dorsolateral BNST (Figure 9) unilaterally from at least 4 sections/animal.

The CRH immunohistochemistry in the PVN was evaluated as the mean intensity using Fiji/ImageJ 1.54 software. We calculated the average intensity as the ratio of the integrated density divided by the area of the PVN from which the background value was subtracted.

### 4.8. Statistical Analysis

All data are presented as the means ± SEMs, with 8–10 animals per group for RT-PCR and 4 animals for IHC. The differences among the experimental groups were analyzed by *t*-test or one-way analysis of variance (ANOVA) followed by Fisher’s LSD post hoc test using SigmaPlotv.11.0 statistical software (Systat Software Inc., Chicago, IL, USA). A value of *p* ≤ 0.05 was considered significant.

## 5. Conclusions

Our results indicate the importance of CRHR2 signaling in anxiety-like behavior and regulation of the stress response. We believe that a better understanding of the role of the CRH system in fear processing and anxiety- and stress-related behavior in psychiatric disorders such as PTSD may pave the way for the use of pharmacological interventions to target the mechanisms leading to the development of PTSD pathology.

## Data Availability

All data presented in this manuscript can be accessed upon request.
